# Dietary addition of a standardized extract of turmeric (TurmaFEED^TM^) improves growth performance and carcass quality of broilers

**DOI:** 10.1186/s40781-018-0167-7

**Published:** 2018-05-28

**Authors:** Johannah NM, Ashil Joseph, Balu Maliakel, Krishnakumar IM

**Affiliations:** R&D Centre, AKAY Flavours & Aromatics Pvt. Ltd, Malayidamthuruthu P.O., Cochin, Kerala 683561 India

**Keywords:** Turmeric, Antioxidant activity, Phytogenics, Growth promoter, Carcass traits, Broiler

## Abstract

**Background:**

Indiscriminate use of antibiotics in livestock and poultry farming has caused emergence of new pathogenic strains. The situation has warrented the development of safe and alternative growth promoters and immunity enhancers in livestock. Herbal additives in animal and bird feed is a centuries-old practice. Thus, the present study investigated the efficacy of a standardized formulation of lipophilic turmeric extract containing curcumin and turmerones, (TF-36), as a natural growth promoter poultry feed additive.

**Methods:**

The study was designed on 180 one-day old chicks, assigned into three groups. Control group (T_0_) kept on basal diet and supplemented groups T_0.5_ and T_1_ fed with 0.5% and 1% TF-36 fortified basal diet for 42 days. Each dietary group consisted of six replicates of ten birds. Body weight, food intake, food conversion ratio, skin colour, blood biochemical analysis and antioxidant status of serum were investigated.

**Results:**

Body weight improved significantly in T_1_ with a 10% decrease in FCR as compared to the control. TF-36 supplementation in T_1_ enhanced the antioxidant enzyme activity significantly (*p < 0.05*) with a decrease (*p < 0.05*) in lipid peroxidation. It also caused a slight yellow skin pigmentation without any change in meat color, indicating the bioavailability of curcumin from TF-36. However, no significant change in the concentration of serum creatinine, total protein and liver enzyme activities were observed, indicating the safety.

**Conclusion:**

In summary, we concluded that TF-36 can be a natural feed additive to improve growth performance in poultry, probably due to the better antioxidant activity and antimicrobial effects contributed by the better bioavailability of curcuminoids and turmerones. Besides, curcuminoids and turmerones were also known to be gastroprotective and anti-inflammatory agents.

## Background

In veterinary medicine, there exists a practice of sub-therapeutic use of antimicrobial growth promoters (AGP) as disease preventive in livestock [1, 2]. However, there are disagreements over the use of AGPs, since it mainly includes a spectrum of human antibiotics whose flagrant and injudicious use in livestock may lead to the emergence of antibiotic-resistant strains and severity of human infections [2]. European Union (EU) had most of the antimicrobial growth promoters banned in 1999 with a strict implementation of the ban from January 2006 onwards [3]. USA is soon to follow the suit with U.S. Food and Drug Administration (FDA) as per the recent Veterinary Feed Directive [4]. Thus, the regulations have imposed a great need for the development and adoption of newer and safer practices for growth promotion and disease prevention in livestock as *natural growth promoters* or *non-antibiotic growth promoters* (NGPs). Most of the current research in this direction is centered around the use of gut conditioners and feed additives employing probiotics, prebiotics, in-feed enzymes, essential oils, herbal extracts and antioxidants [5, 6].

The use of botanicals (maily spices and herbs) in traditional or folk veterinary medicinal system is a widely accepted practice in various countries, with a regional preferences of herbs depending on their availability [7]. Dried powders or extracts or phytochemicals with significant bioactivities (antimicrobial, antioxidant, anti-inflammatory, appetizing, immune-modulatory, and gastroprotective) suitable for use as NGPs in feed have already been classified as ‘*Phytogenics*’ [6, 8]. Phytogenics are natural in origin, categorized Generally Recognized as Safe (GRAS), with desirable pharmacological activities suitable to tackle microbial threats and to promote intestinal health thereby optimizing growth performance and profitability in livestock [6]. Many in vitro and in vivo studies have confirmed the safety and efficacy of phytogenics in animal nutrition. Various kitchen spices and their essential oils and extracts including oregano, ginger, black cumin, turmeric, fenugreek, thyme, coriander, garlic, cinnamon, pepper, clove, rosemary, sage and thyme have already been used singly or in combination as feed additives in animal feeds [9, 10].

Among the various culinary spices, turmeric (*Curcuma longa L*), also known as the *Golden spice*, is of special interest owing to its wide range of beneficial pharmacological effects in supportive of the health and well-being of both animals and humans [11]. Typical composition of dried turmeric rhizomes includes 6-10% (*w*/w) of hexane soluble fat, 3-6% (*w*/w) of volatile oil rich in terpenes and terpenoids, 6-8% (w/w) of proteins, 3-6% dietary fibre and 60-70% carbohydrates [12]. Curcumin or diferuloylmethane, [1,7-bis(4-hydroxy-3-methoxyphenyl)-1,6-heptadiene-3,5-dione], the yellow pigment in turmeric at 3-6% (w/w), has been identified as the major bioacative principle in turmeric with a multitude of effects including antioxidant, anti-inflammatory, antimicrobial, gastroprotective, antiproliferative, antiarthritic and neuroprotective activities [11]. Turmeric oil is yet another source of bioactive molecules, comprising mostly of *ar*-turmerone (60%), curlone (12%), and *ar*-curcumene (6%) along with more than 200 molecules in relatively low levels [13]. Antioxidant, antibacterial, antiviral, antifungal, antihyperlipidemic and wound healing properties of turmeric oil rich in *ar*-turmerones has been demonstrated in various studies [13–16]. Thus, the activity profile and safety of the major lipophilic molecules in turmeric supports its plausible phytobiotic potential. The present study is based on the hypothesis that the dietary addition of curcumin and turmerones can act as a natural antibiotic in poultry feed and may help growth promotion and carcass traits of meat. Thus, we investigated the growth promoting and disease preventive potential of a standardized formulation of turmeric extract containing both curcumin and turmerones (TurmaFEED™; *hereinafter referred to as ‘TF-36’)* as a cost-effective natural antibiotic feed additive for chicken.

## Methods

TF-36 was obtained from M/s Akay Flavours & Aromatics, Pvt. Ltd., Cochin, Kerala, India. Identification, confirmation and quantification of TF-36 has been carried out as per the validated methods, employing high performance liquid chromatography (HPLC) and gas chromatography coupled with tandem mass spectrometry (GC-MS/MS). Analytical reference standards of curcumin (CAS# 458-37-7; purity > 98%), DMC (CAS# 22608-11-3; purity > 98%) BDMC (CAS# 33171-05-0; purity > 95%) and Ar-turmerone (CAS # 532-65-0; purity 95%) were obtained from Sigma-Aldrich, Bangalore, India. HPLC procedure employed Shimadzu model LC 20 AT, with an M20A photo diode array (PDA) detector (Shimadzu Analytical India Pvt. Ltd., Mumbai, India), fitted with a reverse phase C18 column (250 × 4.6 mm, 3 μm) (Phenomenex, Hyderabad, India). *Ar*-Turmerone content was analysed on a gas chromatograph (Agilent 7890B) coupled to with triple quadrupole mass spectrometer (Agilent G7010A). DB-WAX column (0.25 mm, length: 30 m, film thickness: 0.25 u) was used for analysis. The carrier gas was helium and the injector port temperature was 250 °C, transfer line temperature: 280 °C, and ion-source-heating at 230 °C. Interpretation and identification of the fragmentation mass spectrum was carried out by comparison with the Wiley NBS mass spectrum data base. Standardized poultry feed was procured from M/s. Suguna Poultry Feeds, Kerala, India. One day old commercial broiler chicks were purchased from local hatchery (M/s Suguna Foods Limited, Kerala, India).

### Birds and experimental design

The chicks were weighed on arrival (mean weight of 40.21 g) and were randomly assigned to three groups, with each group containing six replicates of 10 birds each. The birds were reared in accordance to the Animal Ethical guidelines and were kept in wire floor cages. The room was equipped with pre-heating facilities and adjustable temperature settings with relative humidity between 65 to 70%, under continuous incandescent white light. The house temperature zones were set at 31-33 °C for the first week and was gradually decreased and reached 24 ± 1 °C by the end of fourth week and then remained constant. Three weight matched replicates were used for each dietary group as per the completely randomized design. Group I (T_0_) was provided with basal diet alone, Group II (T_0.5_) with 0.5% of TF-36 fortified basal diet and Group III (T_1_) with 1% TF-36 fortified basal diet for a period of 6 weeks (42 days). Broilers were provided with the starter basal diet for 14 days and then provided with the finisher diet for remaining 28 days. Both starter and finisher diet were fortified with TF-36 to provide to the experimental groups. Feed and water were provided ad libitum. Nutritional factors of the diet is given in Table 1.

**Table 1:** Nutritional information of the basal diet used

	Nutritional factors	Chick -starter diet	Chick-Grower diet
1.	ME^*^ Kcal/ kg	2915	2915
2.	Crude protein %	17.50	15.00
3.	Fat %	3-4	3-4
4.	Fibre %	3-4	3-4
5.	Lysine %	0.70	0.75
6.	Methionine %	0.40	0.35
7.	Calcium %	1.00	1.00
8.	Total Phosphorus %	0.70	0.60
9.	Vitamin A (I.U.)	4550	4450
10.	Vitamin D (I.U.)	1600	1600
11.	Vitamin E (I.U.)	15.0	15.0
12.	Vitamin K (mg)	1.00	1.00
13.	Choline (mg)	45.0	45.0
14.	Linoleic acid %	1.00	1.00

Animal experiments were in accordance with the protocol approved by the Institutional Animal Ethics Committee, recognized by the Committee for the Purpose of Control and Supervision of Experiments on Animals (CPCSEA), Government of India (Registration No:149/99/CPCSEA).

### Performance measurements

Body weight (BW), feed intake (FI), feed conversion ratio (FCR) and mortality were evaluated as a primary measure of the growth performance of chickens during 42 days of study period. Chickens were weighed individually at days 1, 14 and 42 and feed consumption per cage was recorded on a weekly basis for each replicate in the groups. The FCR (feed intake/weight gain) was calculated as feed consumed per unit of body weight gain. Behavioral changes, mortality and signs of any adverse effects per cage have been checked on a daily basis, during the morning and evening times. Additionally, the average daily weight gain (DWG) was calculated for each group. After the duration of the experiment, (42 days), three birds were randomly chosen from each replicates, sacrificed and their liver, gizzard, heart, pancreas, intestine and kidney were collected, weighed and calculated as a percentage of body weight.

### Blood sampling and biochemical analysis

At the end of the study period (Day 42), blood samples from each replicates were collected from the wing veins into both EDTA and non-EDTA tubes. The blood samples were kept for 2 h at room temperature and centrifuged at 2000 g for 5 min at 4 °C. Separated serum was stored in Eppendorf vials at -20 °C until analysis. Serum levels of superoxide dismutase (SOD), glutathione (GSH), glutathione peroxidase (GPx), and thiobarbituric acid reactive substances (TBARS) were measured using colorimetric methods with a spectrophotometer (UV-2600: Shimadzu Corporation, Tokyo, Japan) as indicated [17–20]. Liver function markers [(Serum glutamic oxaloacetic transaminase or Aspartate aminotransferase (SGOT or AST), serum glutamate-pyruvate transaminase or alanine transaminase (SGPT or ALT)] as well as plasma total protein and serum creatinine were analysed using respective kits provided by M/s Agappe Diagnostics Pvt. Ltd., Bangalore, India (Catalog no. 12005020, 12,005,021, 12,005,022 and 11,009,001).

### Carcass traits and morphometric measurements

At the end of the study (Day 42), birds with nearest average live body weight were randomly selected from each replicate and were deprived of feed for 16 h, then humanely harvested, de-feathered, eviscerated and dressed. The birds were weighed, feet were removed and carcasses were manually eviscerated and the abdominal fat, and giblets (liver, gizzard, heart, kidney, pancreas, intestine) were removed. The organs and meat was washed with saline and were weighed to calculate dressing and edible organs weights as reported earlier [21]. Three broilers per replicate were randomly selected for skin and meat color evaluation.

### Histopathological studies

Liver and heart were dissected out and washed with ice cold PBS, patted dry and fixed in 10% formalin for histopathological examination. Paraffin embedded tissues were sectioned to 5-6 μm thickness and stained with haematoxylin and eosin for histopathological examinations.

### Statistical analysis

The results were subjected to statistical analysis using One-way ANOVA for completely randomized design. Treatment means were compared by Least Significance Difference test through SPSS. *p < 0.05* was considered as statistically significant.

## Results

**Fig. 1:**
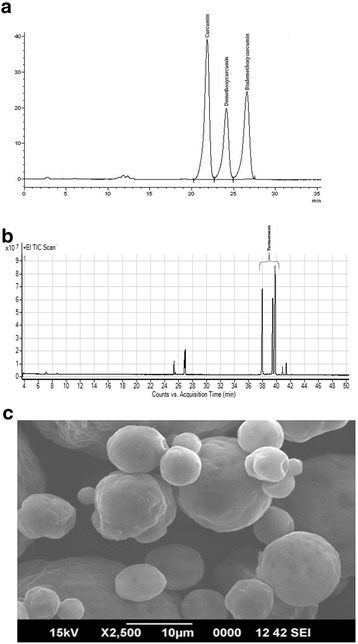
Characterization of TF-36. (**a**) HPLC chromatogram of TF-36 showing curcuminoids. (**b**) Gas chromatogram of the oil fraction of TF-36 indicating α, β and *ar-*turmerones. (**c**) SEM of the powder form of TF-36

TF-36 is a standardized extract of turmeric rhizomes prepared from the solvent extracts employing acetone, hexane, ethylacetate, isopropanol or ethanol, either alone or in combination. TF-36 may be prepared in both liquid and powder form, to allow the easiness in commercial applications. HPLC analysis using standard analytical standards was used for the identification and quantification of curcuminoids in TF-36. It was found that TF-36 contains all the three curcuminoids (curcumin, demethoxycurcumin and bisdemethoxycurcumin) with a total curcuminoids content of 3.1% (*w*/w). GC-MS/MS analysis revealed 6.2% (w/w) of *ar-*turmerones with a total volatile oil content of 13.2% (w/w)(Fig. 1a and b). Scanning electron microscopic studies (SEM) of the  powder form of TF-36 revealed highly encapsulated spherical particles with a large porous surface (Fig. 1c).

### Dosage

The dosage of TF-36 (liquid) or powder was decided on the basis of the readiness by which the chicks consumed the diet. It was found that the most suitable dosage of the liquid form as 0.5% or 1% (*w*/w) of the poultry basal diet, since chicks consumed fortified diet as equally as the basal diet. However, there was some degree of hesitation when fortified at 2% (w/w). In the case of powder form, the chicks were able to consume even at 8 to 10% (*w*/w) fortified level, indicating the flavor and aroma masking efficiency when converted to powder. Since the liquid form of TF-36 can be easily manufactured with relatively low cost of production than the powder form which requires special techniques such as vacuum drying or spray drying, the present study employed the liquid form of TF-36 and fortified the diet at 0.5 and 1% (w/w) levels.

### Growth performance study

**Fig. 2:**
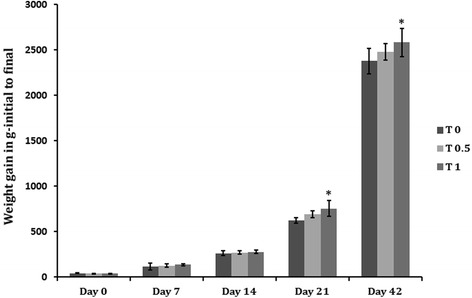
Body weight of the chicks from Day 0 to Day 42. Weight is given in g

The results of the present study showed that TF-36 supplementation at a dose of 1% significantly improved body weight gain (*p < 0.05*) when compared to the normal control group (T_0_) and 0.5% dosage group (T_0.5_). While 0.5% TF-36 fortified diet supplemented group showed no significant changes as compared to the control group (*p > 0.05*), 1% TF-36 supplementation group (T_1_) showed highest total body weight gain (2285 g) as compared to the control group which showed the lowest total body weight (2078 g) (Table 2). The growth performance in T_1_ was also visible from the first week itself and continues to increase progressievely. Figure 2 shows the body weight variation of chicks in a weekly basis.

**Table 2:** Growth performance of the birds

	FCR	Average weight gain (in g)	FI- average of 42 days (in g)
T_0_	1.69	2078.3 ± 139^a^	430.48 ± 6.91^a^
T_0.5_	1.66	2119.6 ± 129^ab^	432.78 ± 5.67^a^
T_1_	1.56	2285.2 ± 154^b^	437.42 ± 9.88^b^

Values are expressed as mean ± SD. Values not sharing a common superscript significantly differ by *p < 0.05*

Food intake (FI) in T1 group (average of 438 g for 42 days) showed a slight deviation from the T_0.5_ group (average of 432 g for 42 days) which was not significant with respect to the control T_0_ group (average of 430 g for 42 days) (Table 2). Supplementation of TF-36 showed a significant decrease in FCR (1.56) in T_1_ when compared with the T_0_ control group (1.69). But, FCR of T_0.5_ (1.68) was not significant (*p > 0.05*) in T_0.5_ (Table 2). In other words, we found 10% increase in body weight with 7.6% decrease in FCR when supplemented with the diet fortified with 1% (*w*/w) of TF-36 (T_1_), whereas these changes were considerably lesser in T_0.5_ with only 2% increase in body weight and 1.7% decrease in FCR, as compared to the normal control (T_0_).

**Fig. 3:**
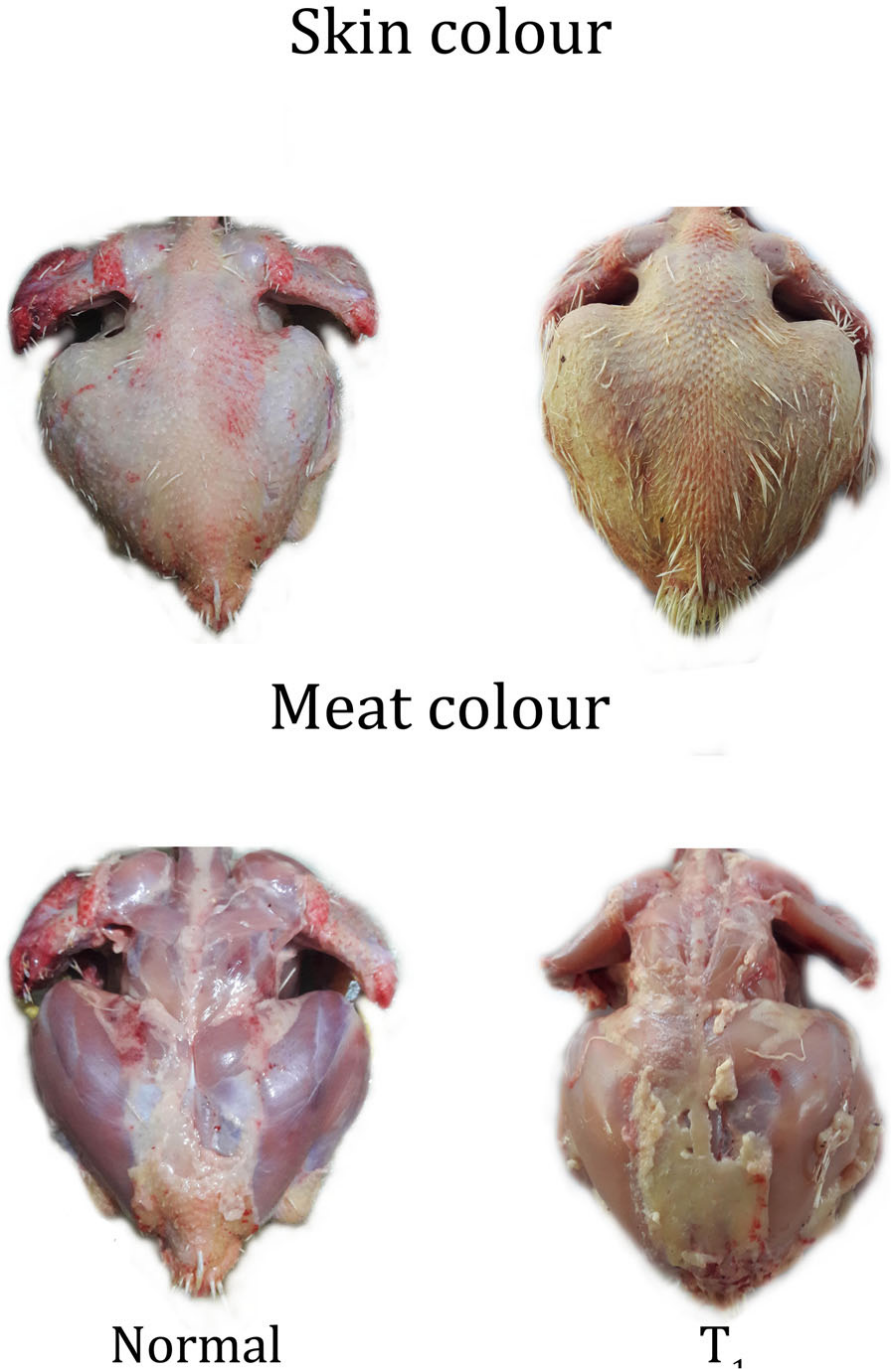
Skin color and meat color of the carcass. Normal pigmentation of skin and meat in T_0_. Yellow pigmentation of skin and normal pigmentation of meat in T_1_

Carcass and visceral organ weight of individual organs were also noted (Table 3). There was a significant increase in the body weight of chicks when supplemented with TF-36 fortified at 1% (w/w) level (T_1_). However, no significant difference was observed in the individual organ weight in relation to their total body weight indicating the absence of any pathology. But the meat of birds, treated with TF-36, showed a visible yellowish pigmentation of the skin as compared to the T_0_, which has no yellowish hue (Fig. 3a and b). None of the groups showed any adverse signs or behavioral changes during the course of the study; except a mortality of 2 chicks in the normal basal diet treated group on day 3 and day 5.

**Table 3:** Weight of the organs on day 42

	Liver (in g)	Gizzard (in g)	Heart (in g)	Pancreas (in g)	Intestine (in g)	Kidney (in g)
T_0_	68.8 ± 1.3	28.5 ± 1.4	11.5 ± 0.8	4.4 ± 0.2	138.1 ± 1.7	15.6 ± 0.8
T_0.5_	69.7 ± 1.5	30.3 ± 1.4	11.9 ± 0.8	4.8 ± 0.2	139.2 ± 2.1	15.9 ± 0.8
T_1_	74.6 ± 2.0^*^	34.1 ± 1.5^*^	14.0 ± 0.5^*^	5.1 ± 0.4^*^	144.2 ± 4.5^*^	16.8 ± 1^*^

Values are expressed as mean ± SD. Values are expressed as mean ± SD. Values not sharing a common superscript significantly differ at *p > 0.05*

### Assessment of endogenous antioxidants

**Fig. 4:**
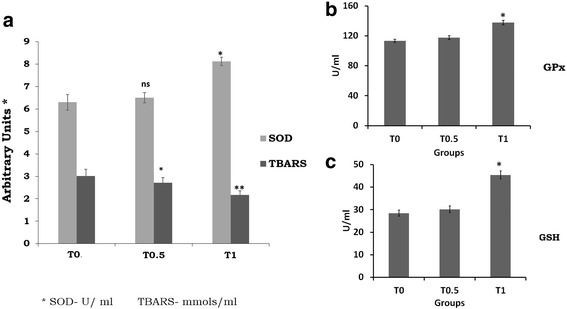
Endogenous antioxidant status. (**a**) Variations in SOD activity and lipid peroxidation as determined by TBARS values, (**b**) Gpx activity and (**c**) GSH activity. SOD and GPx expressed as IU/mL; TBARS as nmols/mL and GSH as mmols/mL. Values are expressed as mean ± SD. Values not sharing a common superscript significantly differ at *p > 0.05*, when the T_1_ or T_0.5_ is compared to T_0_

The effect of supplementation of TF-36 on the antioxidant status of birds is given in Fig. 4. The antioxidant enzyme, SOD showed a significant increase in activity in T_1_ (*p < 0.05*) and those for T_0.5_ was not significant when compared to the control T_0_ (Fig. 4a). GSH and GPx levels were also elevated significantly upon TF-36 treatment as compared to untreated normal chicks (Fig. 4b and c). The extent of lipid peroxidation was measured as TBARS levels and was decreased significantly both in T_0.5_ and T_1_ (*p < 0.05*) groups as compared to the control group T_0_ (Fig. 4a). Thus, an elevation in endogeneous antioxidant levels with a decrease in lipid peroxidation was observed among TF-36 supplemented birds, with significant (*p < 0.05*) variations in T_1_ group as compared to T_0.5_.

**Fig. 5:**
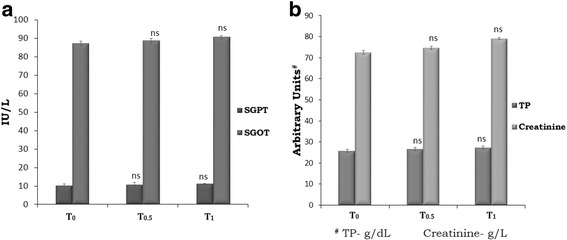
**a** SGOT, SGPT activities and **b** the levels of serum creatinine and total protein. SGOT and SGPT given as IU/mL; total protein as g/dL and creatinine as g/L. . Values are expressed as mean ± SD. Values not sharing a common superscript significantly differ at *p > 0.05*, when the T_1_ or T_0.5_ is compared to T_0_

### Biochemical studies in serum

The studies on total protein revealed no significant difference due to TF-36 supplementation in both the groups T_0.5_ and T_1_ when compared to the control (Fig. 5b). Further analysis of the liver function marker enzymes, SGPT and SGOT also showed no significant variation upon supplementation with TF-36 (Fig. 5a). The relative changes in creatine levels were also remained within the healthy range indicating the absence of toxicity or adverse effect of TF-36 on metabolism (Fig. 5b).

### Histological studies on liver

**Fig. 6:**
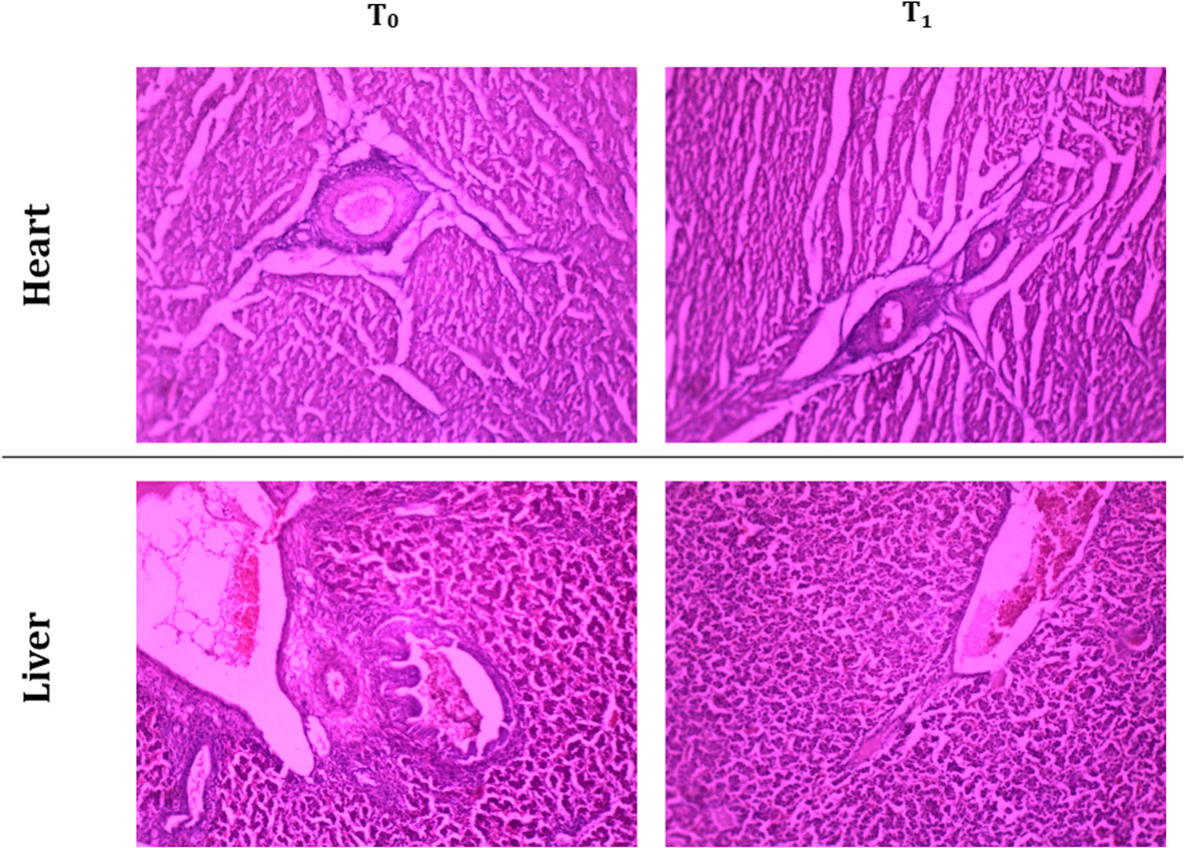
Histopathology of heart and liver. Heart tissue- normal endocardium, normal myocardium and normal pericardium in T_0_ and T_1_. Liver tissue- normal portal triads, normal hepatic veins, intact liver morphology in T_0_ and T_1_

Histopathology of heart showed normal endocardium, myocardium and pericardium both for normal and TF-36 supplemented groups. Histopathology of liver tissues also showed normal portal triads and hepatic veins with normal liver morphology upon supplementation with TF-36 (Fig. 6).

## Discussion

Currently, feed additives for poultry fortified with natural bioactives are being researched aggressively due to the regulation being imposed on the use of AGPs for veterinary purpose. Besides, there is an increased awareness of the risk to human health posed by antibiotics in livestock, propelling research in phytogenic alternatives. Earlier research works suggest the effect of plant extracts in increasing gut microflora and hence better absorption of nutrients with positive results on host nutrition, health, and growth [22]. The digestion stimulating properties and intrinsic bioactivities on animal physiology and metabolism might contribute to the nutritional effects of botanicals [23]. The rhizome of turmeric is a traditional spice and medicine in India for more than 5000 years and the hallmarked yellow colour of turmeric in Indian curries has turned as the signature of a healthy cusine [11, 24]. Curcumin has bbeen researched extensively with more than 5000 publications and is known for its antibacterial, anti-parasitic and antimicrobial actions which render it an effective growth promoter in poultry farming, preventing the incidences of diseases in birds [8]. Turmeric oil, yet another bioactive fraction of turmeric rhizomes with antimicrobial, antibacterial, antifungal and antioxidant properties have not been so far exploited for its phytogenic potential [13–15]. The major terpenes in turmeric oil*,* viz. turmerones were reported to be the reason for its bioactivity [16, 25]. Thus, the present study investigated the natural antibiotic potential of a formulation of turmeric extract with standadised levels of curcumin and turmerones when use as a feed additive in poultry.

Earlier studies on broiler chicken fed with phytogenic blends of coriander, turmeric, thyme have indicated considerable improvement in growth performance, immune indicators and carcass characteristics in both broiler chicken and ducks [26, 27]. The present results were in agreement with these studies and showed significant increase in body weight gain when supplemented with TF-36 at 1% (*w*/w) in chicks. The present study also showed a significant improvement in FCR when treated with TF-36 at 1% level of basal diet. Thus, TF-36 fortified diet implicated a significant effect on growth performance as measured in terms of feed intake, body weight gain and food conversion ratio. The reason for the better growth might be attributed to the antioxidant, anti-inflammatory, antimicrobial, and gastroprotective effect of curcumin and turmerones and their synergic effects [13].

Yellowish skin color in chicken is a desirable characteristic among the chicken consumers in United States and Mexico [28, 29]. Since the yellow colour is known to be imparted by the carotenoids in the diet, regular commercial poultry diet usually fail to provide the colour to birds. Hence, there exist a practice of supplementing carotenoids, either natural or synthetic, to the diet to get them deposited in the skin and fat [30]. Since carotenoids are expensive, a more economical way, especially the natural pigments, to impart yellow color to chicken skin is desirable in poultry production. The present study demonstrated a clear yellow pigmentation to the skin of the chickens treated with TF-36. However, the meat does not show any undesirable colour or flavor characteristics. The yellow pigmentation of the skin is due to the better absorption of the curcuminoids from TF-36. It has already been reported that the oral bioavailability of curcuminoids can be significantly improved upon the co-administration of turmeric oil at significantly high levels of 8 to10% (*w*/w) of the curcuminoids level [11]. The presence of turmeric oil rich in turmerones in TF-36 causes the better absorption of the bioactive yellow pigment curcuminoids, which in turn may enhance the skin colour and quality of the meat by enhancing the antioxidant status and immunity of chicken.

The demand for pre-cooked, refrigerated and/or ready-to-eat food has witnessed a tremendous growth recently. But, preparation of such food products involves processes such as mincing and cooking prior to refrigeration which are known to accelerate lipid peroxidation of meat causing rancidity and therby a deterioration of quality [31]. Poultry meat was reported to be more susceptable to such oxidative deterioration [32]. Addition of synthetic antioxidants to either feed as feed additive or to meat products has been shown to improve the quality of meat [29]. But, reports on the carcinogenicity of synthetic antioxidants paved the way for identification of natural antioxidants as feed additives [33]. Dietary supplementation of rosemary, sage and organo has shown to improve the oxidative stability and reduce lipid peroxidation of raw and precooked broiler meat during refrigeration [34, 35]. Recently, it has been shown that the use of oregano extract as a poultry feed additive improved growth performance and systemic antioxidative capacity of the chicks with an effective inhibition of lipid peroxidation leading to better quality of the meat and bird’s health [36]. The present study demonstrated a significant enhancement in the endogeneous antioxidant status (SOD, GSH and Gpx) and a significant inhibition of lipid peroxidation when supplemented with 1% (*w*/w) of TF-36 as feed additive. Enhancement in the total antioxidant capacity in broilers shall lead to the reduction in oxidative stress and inflammation with improvement in digestibility and hence in growth performance [37]. However, no apparent change was observed in the liver function marker enzymes (SGOT and SGPT) when treated with TF-36. Earlier study had also reported no changes in liver enzymes when supplemented with turmeric powder indicating the absence of adverse effects [26]. Histopathology analysis also confirmed the absence of adverse effects among TF-36 treated birds since no morphological or pathological changes have been observed.

## Conclusion

In summary, the present study showed that the dietary addition of TF-36 at an optimized dosage of 1% (w/w) of feed safely enhanced the growth performance and health status of the chicken. It provided a better body weight gain and food conversion ratio during the study period of 42 days. TF-36 fed chicks showed improved antioxidant effect and better detoxification potential to the birds, along with a significant reduction in lipid peroxidation. It also rendered an yellow colouration to the chicken skin indicating the enhanced bioavailability of curcumin, the bioactive principle responsible for the health beneficial effect of turmeric. Thus, TF-36 might be used as a cost-effective and safe natural growth promoter additive in poultry feed.

## Abbreviations

AGP: Antimicrobial growth promoters
ALT: Alanine transaminase BDMC: Bisdemethoxycurcumin
AST: Aspartate aminotransferase
BW: Body weight
DMC: Demethoxycurcumin
DWG: Daily weight gain
EU: European Union
FCR: Food conversion ratio
FDA: Food and Drug Administration
FI: Food intake
GC-MS/MS: Gas chromatography-tandem masspectrometer
Gpx: Glutathione peroxidase
GRAS: Generally recognised as safe
GSH: Glutathione
HPLC: High performance liquid charomatography
NGP: Natural growth promoters
SEM: Scanning electron microscopy
SGOT: Serum glutamic oxaloacetic transaminase
SGPT: Serum glutamate pyruvate transaminase
SOD: Superoxide dismutase
TBARS: Thiobarbituric acid reactive substance
